# Cyan Emission in Two-Dimensional Colloidal Cs_2_CdCl_4_:Sb^3+^ Ruddlesden–Popper Phase Nanoplatelets

**DOI:** 10.1021/acsnano.1c05684

**Published:** 2021-10-20

**Authors:** Federico Locardi, Margarita Samoli, Alberto Martinelli, Onur Erdem, Debora Vale Magalhaes, Sara Bals, Zeger Hens

**Affiliations:** † Department of Chemistry and Industrial Chemistry, Università degli Studi di Genova, Via Dodecaneso 31, 16146 Genova, Italy; ‡ Physics and Chemistry of Nanostructures group (PCN), Ghent University, Krijgslaan 281, Gent 9000, Belgium; § CNR-SPIN, Corso Perrone 24, I-16152 Genova, Italy; ∥ EMAT and NANOlab Center of Excellence, University of Antwerp, Groenenborgerlaan 171, 2020 Antwerp, Belgium

**Keywords:** Ruddlesden−Popper phase, colloidal nanocrystals, nanoplatelets, emissive materials, metal halides, 2D materials

## Abstract

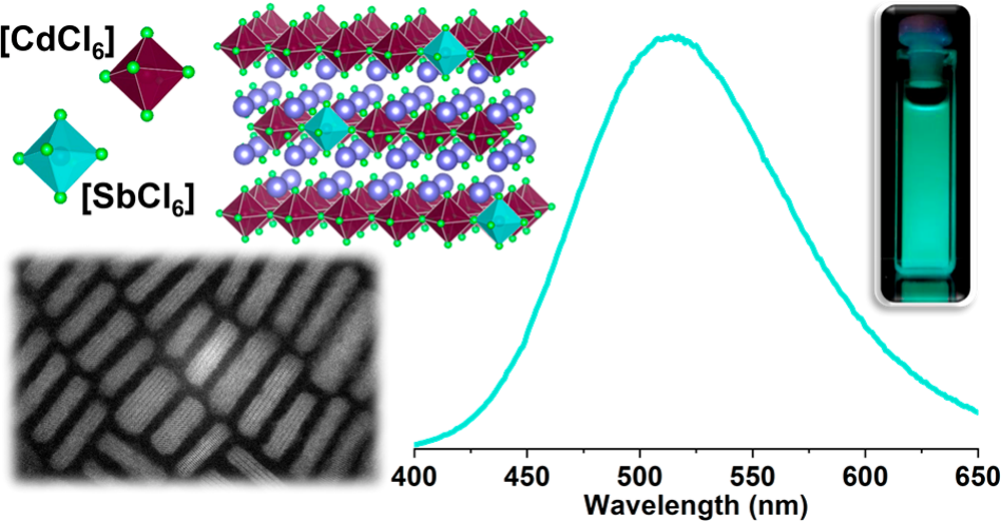

Metal halide perovskites are one of the most investigated materials in optoelectronics, with their lead-based counterparts being renowned for their enhanced optoelectronic performance. The 3D CsPbX_3_ structure has set the standard with many studies currently attempting to substitute lead with other metals while retaining the properties of this material. This effort has led to the fabrication of metal halides with lower dimensionality, wherein particular 2D layered perovskite structures have captured attention as inspiration for the next generation of colloidal semiconductors. Here we report the synthesis of the Ruddlesden–Popper Cs_2_CdCl_4_:Sb^3+^ phase as colloidal nanoplatelets (NPs) using a facile hot injection approach under atmospheric conditions. Through strict adjustment of the synthesis parameters with emphasis on the ligand ratio, we obtained NPs with a relatively uniform size and good morphological control. The particles were characterized through transmission electron microscopy, synchrotron X-ray diffraction, and pair distribution function analysis. The spectroscopic characterization revealed most strikingly an intense cyan emission under UV excitation with a measured PLQY of ∼20%. The emission was attributed to the Sb^3+^-doping within the structure.

## Introduction

Research on metal halides has grown exponentially^1,2^ since the first report on solar cells based on lead halide perovskites^3^ and the preparation of highly emissive colloidal lead halide perovskite nanocrystals. One aspect of this work involves, for example, the substitution of Pb with other metals to form materials with different chemical or physical properties or with better stability than Pb halide perovskite. Noteworthy results that retain the characteristic 3D structure of CsPbBr_3_^4,5^ involve the so-called double perovskites, which are compounds where Pb^2+^ cations are replaced in pairs by a monovalent and a trivalent cation as described by the Cs_2_M^+^M′^3+^X_6_ (X = Cl, Br, and I) formula unit.^5^ Several stable double perovskites (DPs) were prepared both as bulk and nanocrystals, where Na^+^, K^+^, Ag^+^, and so on and In^3+^, Sb^3+^, and Bi^3+^, among others, represent M^+^ and M′^3+^, respectively.^6^ Alternatively, lower dimensional perovskites, in which the metal halide octahedra form 2D structures or isolated 0D units, were explored with (2D) CsPb_2_Br_5_ and (0D) Cs_4_PbBr_6_ as notable examples. In a 2D perovskite, such as CsPb_2_Br_5_, layers consisting of metal halide octahedra and Cs^+^ counterions alternate. In such a structure, the size restrictions that constrain the choice of the counterion in 3D perovskites are greatly relaxed, which enables the formation of hybrid 2D perovskites, in which 2D metal halide layers alternate with layers of organic counterions of various sizes.^7,8^ Such compounds have shown highly interesting optical properties, such as a high photoluminescent quantum yield (PLQY), a tunable emission range, and a narrow emission line widths.

Among all possible arrangements of the metal halide octahedral layers,^7,8^ the so-called Ruddlesden–Popper (RP) phase, stands out due to its chemical diversity, stability, and properties similar to the 3D counterpart.^7,9^ Represented by the formula unit A_2_MX_4_ stoichiometry (A and X = monovalent or bivalent ions; M metal cation, *vide infra*), the RP phase is made from sheets of corner-sharing MX_6_ octahedra with square geometry that are stacked in an ABAB sequence along the *c*-axis. First studied as oxides, such as Sr_2_TiO_4_, in the 1950s by Ruddlesden and Popper,^10^ the same RP structure was later determined for Cs_2_CdCl_4_.^11^ In the 1990s, halide-based RP phases gained attraction due to their interesting optoelectronic properties and are now considered an emerging class of semiconductors materials^7,9^ for solar cells,^12^ optical gain media,^13^ and light-emitting diodes.^14,15^ The most appealing aspect of RP phases is probably the extensive range of organic and inorganic cations that can occupy the A site^7−9,16^ and the several metal and small organic cations, e.g., Cd^2+^,^17−20^ Sn^2+^,^21^ Pb^2+^,^22^ Cu^2+^,^23^ and methylammonium (MA), that can occupy the M site. As demonstrated by the formation of bulk RP phases, this range of compositions creates ample room to adjust chemical properties and tune optoelectronic characteristics.^9^ BA_2_MA_*n*–1_Pb_*n*_I_3*n*+1_ (BA = butylammonium) was, for example, used in solar cells attaining a power conversion efficiency of 16.4%,^12^ while Rb_2_CuCl_2_Br_2_ was found to exhibit an antiferromagnetic behavior promising in spintronic applications.^23^ Cs_2_CdCl_4_ was explored as an X-ray scintillator material that can be made luminescent through Sb^3+^ or Tl^+^ doping.^24^ Here, Cs_2_CdCl_4_:Sb^3+^ particularly stood out by having only a single emission band, with a maximal intensity at 516 nm.

Despite progress in the formation of bulk materials, the use of RP phases as an optoelectronic material is still hampered by the difficulty to obtain phase-pure films that exhibit a spatially homogeneous composition and crystal structure.^9^ In this regard, the use of presynthesized colloidal nanocrystals^25−28^ or nanoplatelets as starting materials is a promising alternative.^29−32^ Even so, few studies have addressed the formation of RP phases as colloidal nanocrystals. To the best of our knowledge, only the formation of Cs_2_PbCl_2_I_2_ and Cs_2_PbCl_2_I_2_:Mn has been reported in literature.^33−35^ In more detail, Cs_2_PbCl_2_I_2_ exhibited a rather weak blue emission, while Mn^2+^ doping resulted in an intense orange emission (PLQY of 16%). Still, such a limited range of RP phases is insufficient for exploring the distinct chemical and physical characteristics of these materials when synthesized as colloidal nanocrystals, both in comparison with the corresponding bulk compound and in view of the eventual application of RP phases for optoelectronics.

Here, we report on the colloidal synthesis of Cs_2_CdCl_4_:Sb^3+^ as lead-free metal halide colloidal RP phase. This RP phase is a useful test compound, given the well-defined emission characteristics observed in the bulk counterpart. We show that through a strict control of the synthesis parameters, especially the ligand amount and ratio, a phase pure nanocrystal can be formed consisting of Cs_2_CdCl_4_:Sb^3+^ nanoplatelets (NPs). The nanocrystal morphology and crystal phase are established through an extended structural characterization based on X-ray powder diffraction (XRPD), pair distribution function analysis (PDF) using synchrotron radiation, and transmission electron microscopy (TEM). We demonstrate that the synthesis method yields NPs with a well-defined size and morphology. Moreover, we retrieve an intense cyan emission with a PLQY of ∼20% that we ascribe to s–p transitions in Sb^3+^, not unlike bulk Cs_2_CdCl_4_:Sb^3+^.^24^ Interestingly, we find that the RP structure of Cs_2_CdCl_4_ NPs is distorted along the *c*-axis. This ground-state anisotropy questions the assignment of the multiplet structure of the s–p transitions to a dynamic Jahn–Teller effect. We thus conclude that colloidal synthesis methods can give access to a diversity of RP phases, apart from lead metal halides. Moreover, exploring the chemical and physical properties of such nanocrystals will enhance the fundamental understanding of RP phases and make possible to use these 2D compounds as an optoelectronic material.

## Results and Discussion

**Figure 1:**
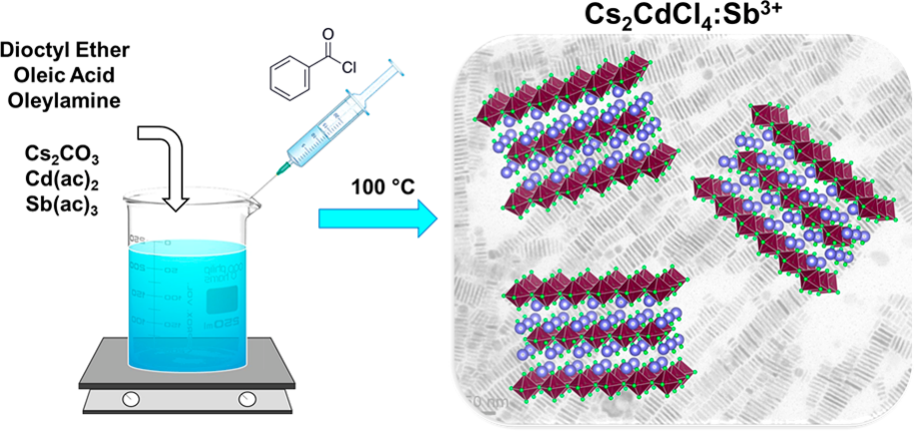
Schematic representation of the Cs_2_CdCl_4_:Sb^3+^ NPs synthesis.

For the synthesis of undoped and Sb^3+^-doped Cs_2_CdCl_4_ nanoplatelets (Figure 1), we modified a protocol that was used for the preparation of the Cs_2_Ag_1–*x*_Na_*x*_InCl_6_ double perovskite nanocrystals.^36^ We mixed Cs_2_CO_3_, Cd(II)-acetate and Sb(III)-acetate with dioctyl ether and added oleic acid and oleylamine as possible ligands. To form NPs, this mixture was heated, and benzoyl chloride was swiftly injected as a halide precursor at 100 °C. The reaction was rapidly arrested by quenching in an ice bath. Importantly, we found that small variations in the experimental conditions, such as ligand ratio, temperature, and Sb(ac)_3_ amount, led to strong variations in the synthesis outcome, ranging from a more polydisperse end product to the dominant formation of CsCl (see Supporting Information and Figures S1 and S2). The synthesis protocol optimized for forming phase pure, monodisperse Cs_2_CdCl_4_:Sb^3+^ colloidal nanoplatelets, is reported in the “Methods” section.

**Figure 2:**
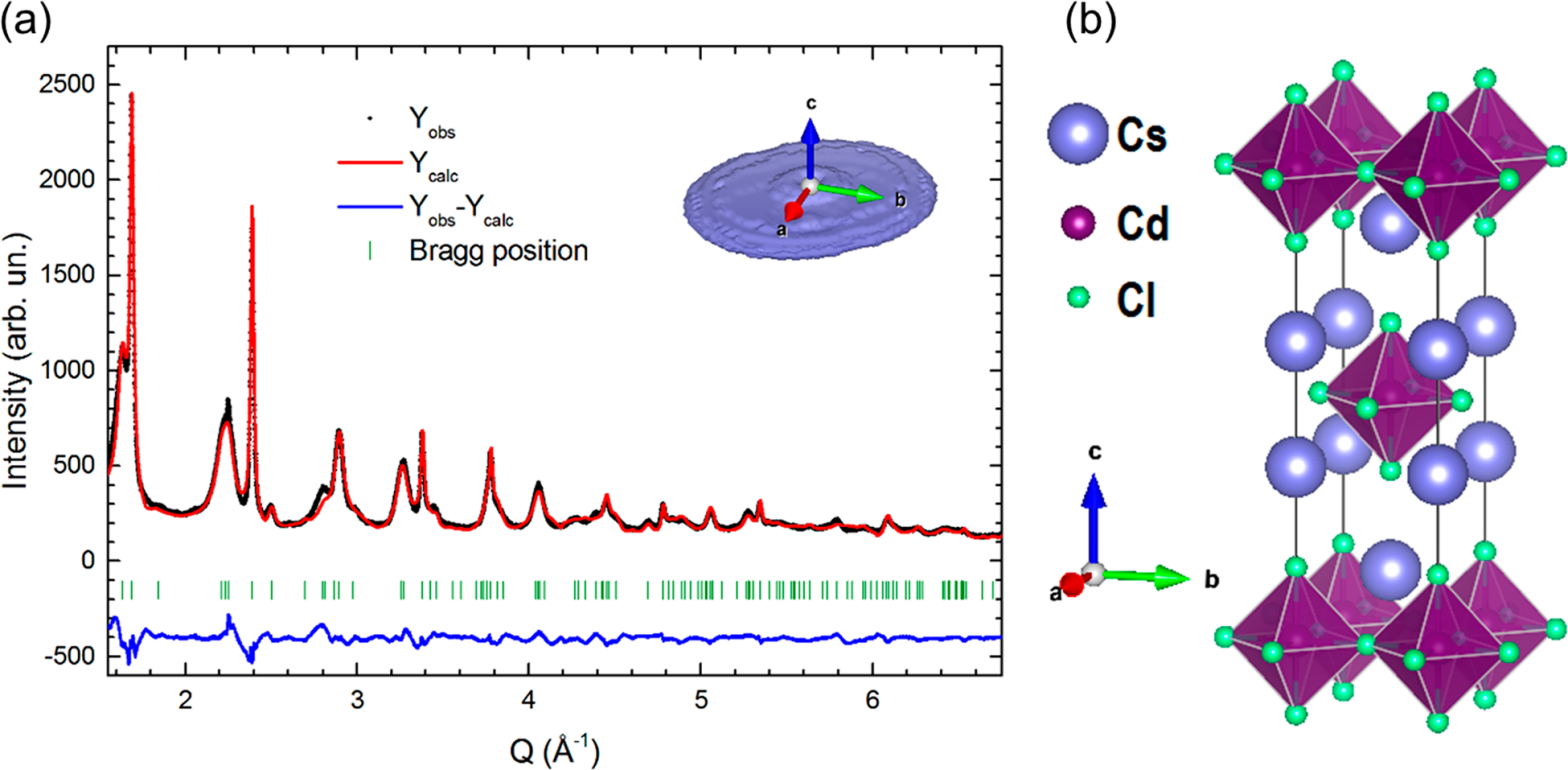
(a) Rietveld refinement for Cs_2_CdCl_4_ (synchrotron XRPD data; the inset shows the average morphology of the coherent diffraction domains as obtained by diffraction line broadening analysis). (b) Representation of the Cs_2_CdCl_4_ crystal structure.

The structural and microstructural characterization of the prepared samples was carried out by XRPD and PDF analysis using synchrotron radiation (see the “Methods” section). Rietveld refinement (Figure 2a) reveals that the sample is fully composed of Cs_2_CdCl_4_:Sb^3+^ and crystallizes in the tetragonal *I*4/*mmm* space group (; no. 139; Figure 2b). Importantly, the XRPD data collected for the undoped sample does not show any relevant structural difference with respect to the doped compound (Figure S3). The calculated lattice parameters for Cs_2_CdCl_4_:Sb^3+^ are 5.2568(1) and 16.876(1) Å for *a* and *c*, respectively (Table S1). As evidenced by the XRPD pattern, the diffraction lines of different families of planes display a strongly different broadening. Such variations indicate a sizable shape anisotropy. An in-depth analysis indicates that coherent diffraction domains are characterized by a lamellar shape (Figure 2a, inset). The particle size was subsequently determined by modeling the experimental XRPD pattern profile with Debye function analysis using the software DIANNA.^37^ Different unit cells along *ab* and *c* were tested, and the best fit was obtained considering a particle dimension of 44 × 44 × 3 unit cells corresponding to a particle dimensions of 23.1 × 23.1 × 5.1 nm^3^ (Figure S4).

**Figure 3:**
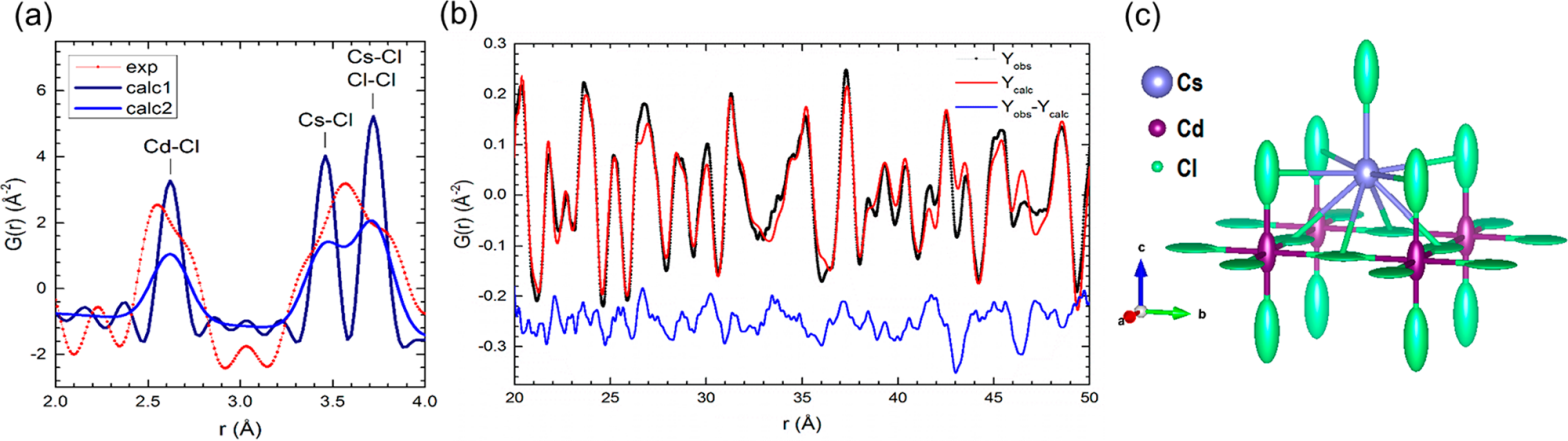
(a) Experimental *G*(*r*) function of Cs_2_CdCl_4_:Sb^3+^ in the low-*r* region compared with the corresponding calculated *G*(*r*) functions in the ideal case (*calc1*) and by taking into account the experimental resolution (*calc2*). (b) fitting of the experimental *G*(*r*) function in the 20 < *r* < 50 Å range. (c) Arrangements of Cl atoms around Cs and Cd in the Cs_2_CdCl_4_ crystal structure; atoms are drawn as displacement ellipsoids (90% of probability) evidencing a fluctuation along the *c*-axis.

For a more detailed view of the crystal structure, PDF analysis was performed. The analysis of the experimental *G*(*r*) function in the low-*r* region reveals a relevant distortion of the local structure of Cs_2_CdCl_4_:Sb^3+^(Figure 3a) as evidenced by the bimodal distribution of the nearest Cd–Cl bond distances (broad split peak at ∼2.6 Å).

Therefore, the *I*4/*mmm* structural model is inadequate to fit the data in this low*-r* region. For larger distances, (20–50 Å), the *I*4/*mmm* structural model gives a better fit, even if significant deviations are apparent as the distortions propagated even at larger distances (Figure 3b). Such deviations from the structural model are likely produced by a dynamic distortion characterizing the octahedral framework of the Cs_2_CdCl_4_ structure, as also evidenced by the oscillations observed at high *Q* in the total scattering *S*(*Q*) function curves (see Figure S5). By analyzing the anisotropic Debye–Waller parameters obtained after data fitting, it is evident that such structural distortion can be mainly ascribed to the dynamic fluctuations affecting the axial Cl position.^38,39^ Drawing atoms as displacement ellipsoids, see Figure 3c, notable fluctuations can be discerned for both Cd and axial Cl atoms along the *c-*axis. These displacements can be related to the local scale distortion observed in the low-*r* region of the experimental *G*(*r*) function (Figure 3a). Considering the crystal structure constraints, it is reasonable to hypothesize that Sb^3+^ substitutes Cd^2+^; indeed, the optical properties of the material resemble the presence of [SbCl_6_] octahedra (*vide infra*). Recently, we showed that the introduction of a trivalent lanthanide in the CsPbBr_3_ induced the filling of halides vacancies rather than Cs^+^ expulsion.^40^ In point of fact, it is known that metal halides are often anion deficient, i.e., present several vacancies in the halide sites.^38^ Thus, the Cd^2+^ → Sb^3+^ is balanced by filling of a Cl^–^ defect.

**Figure 4:**
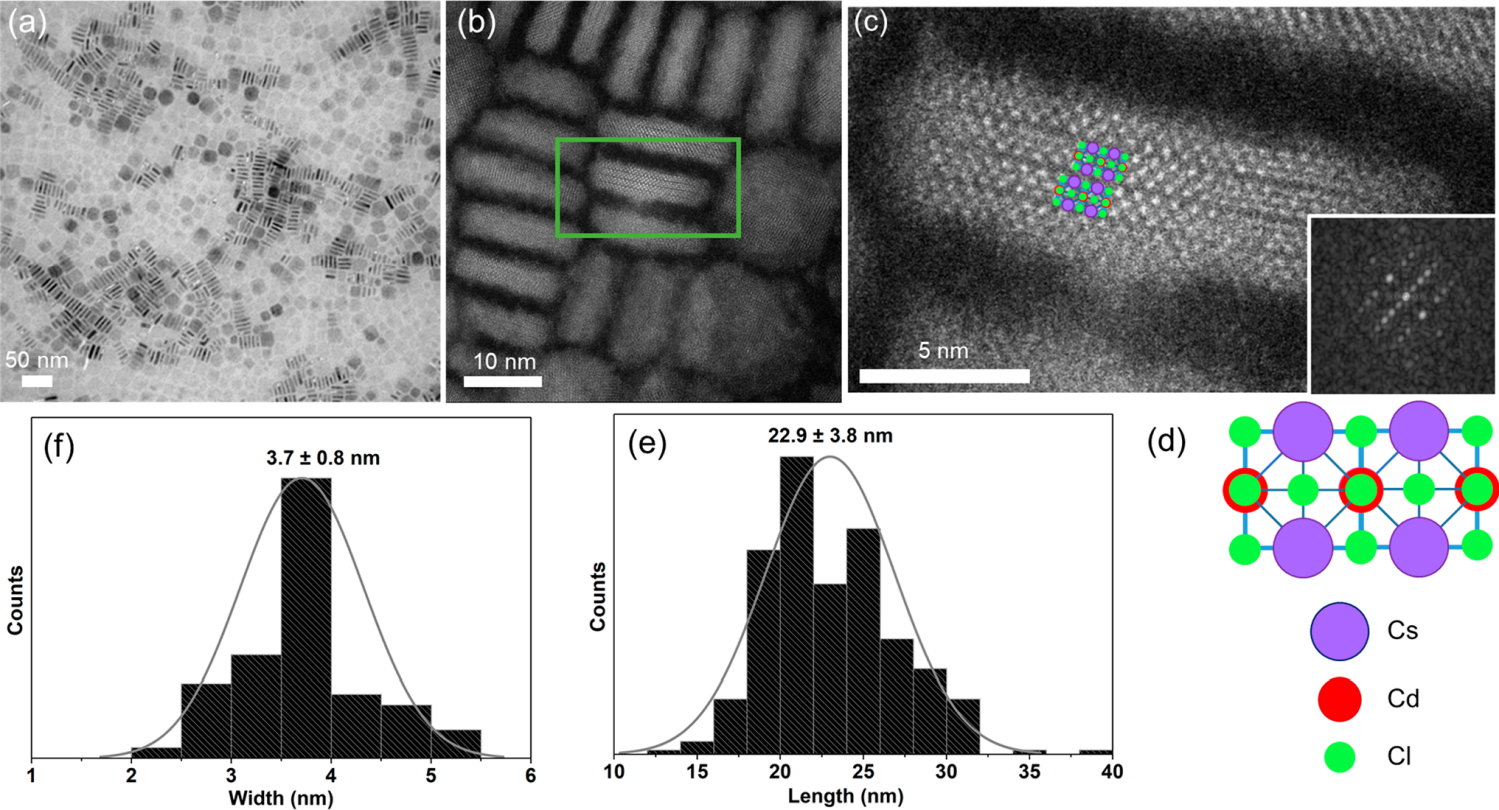
(a) Bright-field TEM and (b) HAADF-STEM image overview of the Sb^3+^ doped Cs_2_CdCl_4_ nanoparticles. (c) Magnified view of the nanoparticle inside the green rectangle present in (b), alongside the corresponding diffractometer pattern (inset). The corresponding FFT pattern can be indexed as the [100] zone of the *I4/mmm* tetragonal space group. (d) Expected atomic columns in zone [100] that match with the observed atomic columns. (e) Length and (f) width size distribution profile with an averages of 22.9 ± 3.8 nm and 3.7 ± 0.8 nm, respectively.

The main findings of a transmission electron microscopy (TEM) study are represented in Figure 4. As can be seen in Figure 4a, the optimized synthesis protocol yields regular nanoplatelets (NPs) with a length of 22.9 ± 3.8 nm and a thickness of 3.7 ± 0.8 nm. These dimensions are consistent with the predictions based on the XRPD data. Interestingly, the NPs have a natural tendency to stack along the *ab* top surface, self-assembling into long chains (Figure S6). No significant differences were detected between the undoped and doped samples, suggesting that the introduction of Sb has no influence on the final morphology (Figure S3). High-angle annular dark-field (HAADF) scanning TEM imaging confirmed the crystallinity of the NPs and gave no evidence of defects along the nanoplatelets (see Figure 4b,c). The TEM image agrees with the Cs_2_CdCl_4_ structure and shows the characteristic RP phase formed by the staggered stacking of Cs_2_CdCl_4_ layers (Figures 2b, 4c,d, S7). The number of observed layers is compatible with particles formed by 2.5–3 cells along *c* and in good agreement with the average thickness and the modeling of the XRPD data. The Sb amount was quantified by X-ray fluorescence (XRF) analysis, giving an average Sb amount equal to 1.4%.

**Figure 5:**
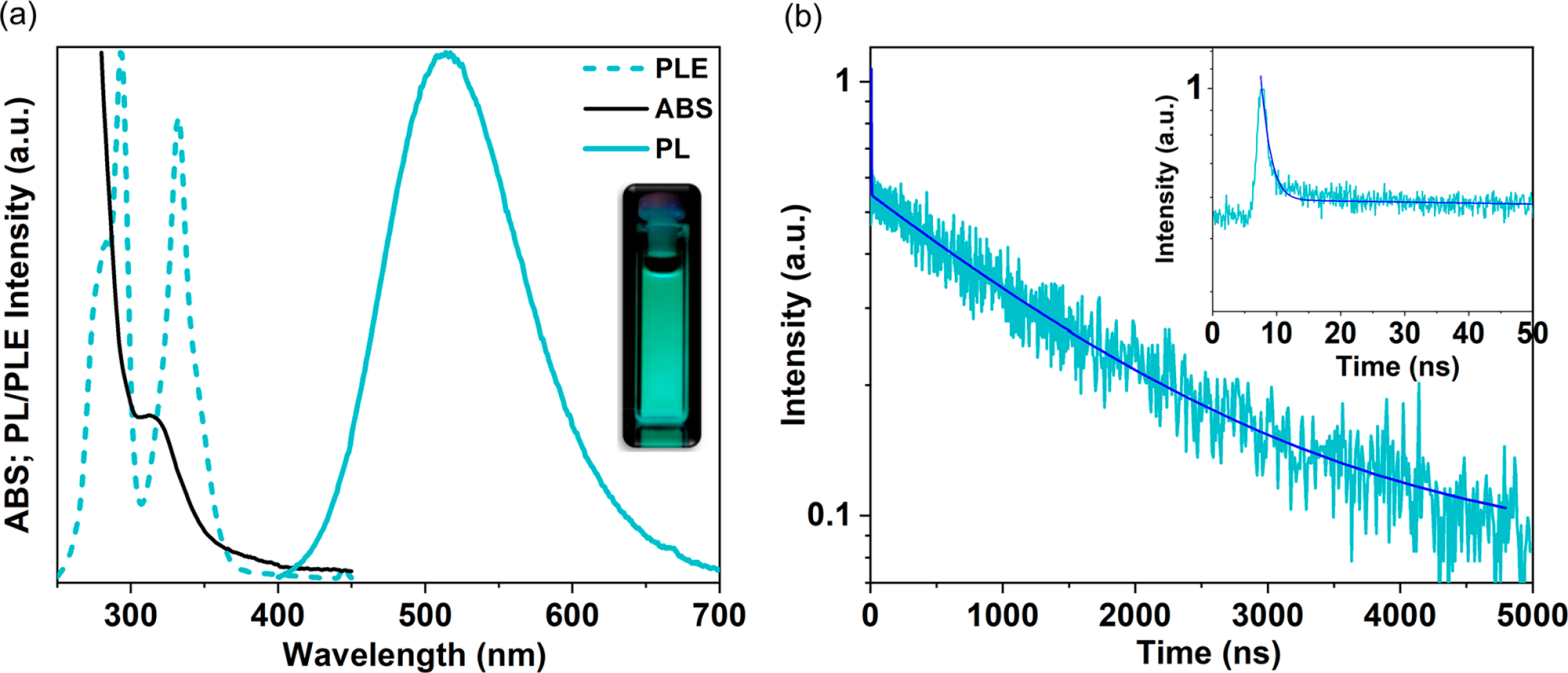
(a) ABS (solid black), PL (solid teal), and PLE (dashed teal) spectra of Cs_2_CdCl_4_:Sb^3+^ NPs. Inset: photo of the sample emission under UV illumination. (b) Time-resolved PL of Cs_2_CdCl_4_:Sb^3+^ dispersed in hexane collected at the emission maximum (510 nm), together with the biexponential fitting curve (dark blue).

We also studied the optical properties of the NPs. Figure S8 displays the absorbance (ABS) spectrum of the Cs_2_CdCl_4_:Sb^3+^ NPs as well as that of undoped Cs_2_CdCl_4_ NPs. The undoped NPs show no absorption up to ∼280 nm, consistent with the recent report by Holder et al.^17^ The Sb^3+^ addition strongly influences the optical properties by introducing transitions at lower photon energy (Figures 5a and S8). The ABS spectrum shows that the Cs_2_CdCl_4_:Sb^3+^ NPs are completely transparent up to 400 nm, followed by a pronounced feature peaking at ∼320 nm. It is worth noting that similar ABS behavior was observed both in the Sb-based, e.g., Cs_3_Sb_2_Cl_9_ and Cs_4_CdSb_2_Cl_12_,^41,42^ and Sb-doped perovskites, e.g., Cs_2_MInCl_6_:5%Sb^3+^ (M= Na and K).^43^ Interestingly, the feature at ∼320 nm observed in Cs_2_CdCl_4_:Sb^3+^ NPs sample is quite sharp and pronounced, which may be ascribed to the small thickness of the NPs. At ∼280 nm, an additional line appears only in the doped NPs (Figure S8a), which we tentatively ascribe to the C band of Sb^3+^ (*vide infra*). Below 275 nm, the absorption is ascribed both to the matrix^17^ and to the ligands attached on the surface (Figure S8b). Upon excitation at λ > 250 nm we did not detect any emission for the undoped Cs_2_CdCl_4_ NPs, consistent with Sakai et al.,^24^ who reported an emission only exciting in the VUV region using synchrotron radiation. However, Cs_2_CdCl_4_:Sb^3+^ NPs shows a broad emission under UV excitation (fwhm 103 nm) which is slightly blue-shifted from the bulk^24^ and centered at 510 nm, resulting in a cyan color (Figure 5a).^44^

The photoluminescence excitation (PLE) spectrum consists of two different bands around 290 and 340 nm (Figure 5a). Typically, Sb^3+^ possesses an allowed ^1^S_0_–^1^P_1_ and a partially allowed ^1^S_0_–^3^P_1_ transition named C and A bands, respectively,^45−49^ which reportedly split into a triplet and a doublet due to a pseudo-Jahn–Teller effect.^43,50,51^ The PLE spectrum of Cs_2_CdCl_4_:Sb^3+^ follows this predicted behavior, with the A band evidenced by the features at ∼350 and 333 nm and the C band evidenced by the signals at 293 and ∼280 nm (Figure S9), mostly resembling the bulk material.^24^ The third peak of the triplet, which is expected to be around 270 nm, might be partially shadowed by other features. Interestingly, the PDF analyses shown here (Figure 3) evidenced that a significant uniaxial distortion at local scale is already present in the unexcited NPs. Consequently, we hypothesize that the multiplet structure originates from the intrinsic complex structural dynamics that affect these materials,^52^ rather than a pseudo-Jahn–Teller effect. Further analyses are needed to clarify this, which are out of the scope of this work.

Importantly, the PLE spectrum can be superimposed on the ABS spectrum (Figure S9) and resembles the one observed for Cs_2_CdCl_4_:Sb^3+^ single crystals,^24^ well demonstrating the two different transitions. Moreover, the PL/PLE properties of Cs_2_CdCl_4_:Sb^3+^ and Sb^3+^-DPs, e.g., Cs_2_NaInCl_6_:Sb^3+^,^43^ are similar, confirming that the Sb^3+^ suballocates in the Cd^2+^ position, that has the same octahedral symmetry of [SbCl_6_]^3–^ in DPs. The PL emission perfectly approximates what is observed in the Cs_2_CdCl_4_:Sb^3+^ single crystal,^24^ highlighting the origin from self-trapped excitons (STE), i.e., with no evidence of a quantum confinement effect induced by the size reduction. PLE scans were also taken at different emission wavelengths, and the respective plots were superimposed upon each other (Figure S10). The matching spectra are a good indication that the emission originates from the same excitation host. The measured emission intensity is quite high evidenced by a PLQY of 20 ± 5%. Decreasing or increasing the amount of Sb precursor leads to a reduction in PLQY, indicating that we reached the optimal doping level at 1.4% before having a self-quenching phenomenon^43^ (Figure S2). A PLQY of 20% is comparable to that of cyan-emitting lead halide nanocrystals (LHCs)^53^ and is promising for the implementation of RP phases in optoelectronic devices.^44^ Importantly, different from LHCs which suffer from halide migration that induces a variation in the optical emission,^53−55^ we did not observe any changes in the PL position of Cs_2_CdCl_4_:Sb^3^ even after several months of storage (Figure S11).

Time-resolved photoluminescence (TRPL) measurements were subsequently conducted to measure the photoluminescence lifetime (Figure 5b). The PL decay consists of a fast and slow component, as was similarly reported for the respective single-crystal phase.^24^ A biexponential curve was fitted and from the resulting equation the values acquired are 1.28 ± 0.04 ns and 1625.9 ± 26.9 ns, respectively. The existence of these two components could also be attributed to two different recombination routes from the excited states of Sb^3+^_._ Reisfeld et al. reported from the excitation spectra of Sb^3+^-doped glass substrates the transitions ^3^P_0_ → ^1^S_0_ for the fast decay and ^3^P_1_ → ^1^S_0_ for the slow decay as possible recombination routes.^56^ This recombination mechanism is further supported by the findings of Noculack et al., who worked on Sb^3+^-doped Cs_2_MInCl_6_ (M = Na and K) double perovskites and could also be operational in Cs_2_CdCl_4_:Sb^3+^.^43,56^

Finally, in order to investigate the stability of the cyan-emitting NPs, we stored the sample in a vial under air for 6 months. The TEM images showed that the nanoplatelets are still present but with a reduced homogeneity (Figure S11a); however, the samples still preserve the optical properties (Figure S11b) albeit with a lower emission intensity. The crystal structure is also preserved, and no secondary phases can be detected by the XRPD (Figure S11c).

## Conclusions

In conclusion, we successfully prepared the fully inorganic Ruddlesden–Popper Cs_2_CdCl_4_:Sb^3+^ nanoplatelets. Through strict control of the synthesis parameters, especially the ligand ratio, colloidally stable nanoplatelets with a length and thickness distribution of 22.9 ± 3.8 nm and 3.7 ± 0.8 nm, respectively, were obtained. A detailed structural investigation confirmed the phase purity and identified significant distortion at the local scale. We find that Sb^3+^-doping introduces additional bands in the absorption spectrum and leads to a cyan emission. Both features can be assigned to s–p transitions in Sb^3+^, where the distortion of the ground-state structures questions the assignment of the absorption band splitting to a dynamic Jahn–Teller effect. The cyan emission exhibits a double exponential decay, characterized by a fast (∼1.3 ns) and a long (∼1626 ns) component. The PLQY was quantified as ∼20%. The similar structural and optical properties of bulk and nanoplatelet Cs_2_CdCl_4_:Sb^3+^ highlight the potential of colloidal chemistry methods to study the full diversity of RP phases, through halides tuning, metal alloying, e.g., Mn^2+^, Zn^2+^, and so on, the replacement of Cs^+^ by alternative counterions and the introduction of various dopants, such as Bi^3+^ or Mn^2+^. Such studies will greatly improve our fundamental understanding of the chemical and physical properties of RP phases and make possible the application of these compounds as an optoelectronic material.

## Methods

### Chemicals

Cesium carbonate (Cs_2_CO_3_, 99.9%, Sigma-Aldrich), antimony acetate (Sb(ac)_3_, 99.99%, Sigma-Aldrich), cadmium acetate dihydrate (Cd(ac)_2_·2H_2_O, 98% analytical grade, Acros Organics), dioctyl ether (DoE, 99%, Sigma-Aldrich), hexane (Chem Lab NV), degassed oleylamine (OLAm, 80–90% C18 content, Acros Organics), oleic acid (OLAc, 90%, Alfa Aesar), and benzoyl chloride (Bz-Cl, ACS reagent, Sigma-Aldrich) were used without further purification.

### Synthesis of Cs_2_CdCl_4_:Sb^3+^ Nanoplatelets

The reaction was performed under atmospheric conditions. In a 20 mL glass vial, Cs_2_CO_3_ (0.125 mmol), Sb(ac)_3_ (0.25 mmol), and Cd(ac)_2_·2H_2_O (0.125 mmol) were weighed. Next, 4 mL of DoE was subsequently added, along with ligands OLAm (1.4575 g) and OLAc (1.0403 g). It should be stressed here that from our experience the OLAm/OLAc ratio is a crucial factor in order to successfully reproduce the sample; therefore, the OLAm amount might need adjustment (slight increase or decrease) depending on the grade quality of the OLAm used. In the meantime, a syringe of Bz-Cl (400 μL) diluted in degassed DoE (400 μL) was prepared in the glovebox. The vial with the precursor salts was quickly heated at 120 °C using a hot plate and an aluminum block; subsequently, the temperature was raised until 160 °C in about 8 min. The vial was then lifted from the block and left to naturally cool down under stirring until an injection temperature *T* = 100 °C was reached. Bz-Cl was then injected, and the vial was immediately quenched in an ice bath. For purification, the sample was centrifuged at 4500 rpm for 5 min. The supernatant was discarded, and the white precipitate was centrifuged again for an additional 2 min to remove as much of the liquid as possible. The sides of the vial were then wiped with a lint-free tissue. The precipitate was redispersed in 3 mL of hexane, and the vial was centrifuged at 3000 rpm for 5 min. The supernatant was kept while the precipitate was discarded.

### Synthesis of Cs_2_CdCl_4_ Nanoplatelets

The synthesis follows the procedure used for the Sb^3+^-doped Cs_2_CdCl_4_ except for the Sb(ac)_3_ that was not added in the reaction mixture.

### Optical Spectroscopy

The UV–visible absorption (ABS) spectra were recorded using a PerkinElmer Lambda 365 spectrophotometer. The photoluminescence (PL), photoluminescence excitation (PLE), and time-resolved photoluminescence decay (TRPL) scans were collected using an Edinburgh FLSP920 spectrometer in 90° geometry. The samples were prepared in Hellma quartz cuvettes (10 mm light path) through dilution of the colloidal suspension in 3 mL of hexane. For the PL and PLE, the samples were excited at 330 nm using a continuous Xe300 lamp. For the TRPL decay traces, the samples were excited using an Edinburgh EPLED-330 (pulse width: 871.7 ps) over a range of pulse repetition rates (5, 0.5, and 0.2 MHz) in order to capture both decay components with good temporal resolution. A long pass filter (transmission >90% at λ > 390 nm) was placed between the sample and the detector to prevent the collection of the excitation pump. The decay trace is fitted to a biexponential curve. Quantum yield (PLQY) measurements were performed using the integrating sphere designed for the FLSP920 spectrometer. The same cuvette (capped with a PTFE stopper) was used for measuring the blank (3 mL of hexane) and the sample (OD < 0.3). The sample was excited at 330 nm with the excitation slit set at 10 nm. The sample and reference scatters were collected in the 310–350 nm range; the emission was collected in the spectral range of 400–700 nm. The spectra were recorded using the following parameters: step of 0.5 nm, dwell time per step of 0.2 s, 2 repetitions, and detection slit at 2 nm. The same long pass filter was placed between the sphere and the detector for removing the second harmonic of the excitation source. The collected spectra were automatically corrected by the software, and the PLQY was calculated integrating the scattered and emitted photons by the blank and the reference. The PLQY quantification was performed in duplicate on two different batches.

### Transmission Electron Microscopy (TEM) Characterization

Bright-field TEM (BF-TEM) imaging was performed on a JEOL JEM-1011 microscope equipped with a thermionic gun operating at 100 kV accelerating voltage. High-resolution high-angle annular dark-field scanning transmission electron microscopy images were acquired with a probe-corrected cubed Thermo Fisher Scientific Titan microscope operating at 300 kV with a probe semi convergence angle of 20.5 mrad. Size distribution was calculated using ImageJ with the MorphoLibJ plugin.

### X-ray Powder Diffraction (XRPD) Measurements

Data for the undoped and aged samples were collected using a Siemens D5000 equipped with a Cu Kα X-ray tube operating at 0.154 nm. The specimens were prepared by drop-casting 800 μL of the NPs suspension onto a 2 cm glass slide. The data was collected between a 2θ range of 5–110° with a step size of 0.02° and a step time of 10 s/step. The collected pattern was then compared with reference patterns attained from the ICSD database, provided by FIZ Karlsruhe GmbH.

### Synchrotron X-ray Powder Diffraction (XRPD) and Pair Distribution Function (PDF) Analyses

Data on the Sb-doped sample were collected at the ID22 of the European Synchrotron Research Facility (ESRF) in Grenoble, France. Data was acquired at 300 K. For XRPD, a wavelength λ = 0.3540 Å was used. Structural refinement was carried out according to the Rietveld method^57^ using the program Fullprof^58^ and a file describing the instrumental resolution function. At this scope, a standard LaB_6_ sample was analyzed using the same experimental conditions. In the final cycle, the following parameters were refined: the scale factor; the zero point of detector; the background (linear interpolation between a set of fixed points); the unit cell parameters; the atomic site coordinates not constrained by symmetry; the atomic displacement parameters; the anisotropic size parameters. PDF data were collected using a wavelength λ = 0.2067 Å. Data from an empty borosilicate capillary were collected to subtract the container scattering; moreover, a standard LaB_6_ sample was analyzed using the same experimental conditions in order to describe the experimental resolution effects.

Reduction of the total scattering data to obtain *G*(*r*) was achieved by the PDFgui software using *Q*_max_ = 25.0 Å^–1^.^59^ Full-profile fitting of the *G*(*r*) function was carried out using the PDFgui software,^60^ and the parameters obtained from the Rietveld refinements as starting structural models. In the last cycle of the fitting the following parameters were refined: the scale factor; the structural parameters; the atomic positions not constrained by symmetry; the anisotropic atomic displacement parameters according to the space group symmetry; and the dynamic correlation factor. Moreover, the parameters describing the experimental resolution effects (*Q*_damp_ and *Q*_broad_) were fixed to the values refined using the standard LaB_6_ sample.

### X-ray Fluorescence (XRF) Measurements

The XRF measurements were collected using a Rigaku NEX CG EDXRF, equipped with a Pd anode X-ray source with 50 W max power at 50 kV max voltage. The spectrum data was collected from Al, Cu, and RX9 secondary polarization targets. The measurement was performed under a 1 bar He atmosphere. For the sample preparation, the colloidal suspension was drop-cast onto stretched prolene films mounted in 31 mm diameter plastic cups and left to dry before measuring. The qualitative and quantitative analysis was performed using the accompanying NEX software.

## Supporting Information Available

Synthesis optimization conditions; structural and X-ray total scattering data; additional TEM and HRTEM images; ABS and PLE of the Cs_2_CdCl_4_:Sb^3+^; XRPD, TEM image, and ABS of the undoped Cs_2_CdCl_4_; XRPD, TEM, ABS, PLE, and PL of Cs_2_CdCl_4_:Sb^3+^ after 6 months (PDF)
