# Supplementary material for: Cyan Emission in Two-Dimensional Colloidal Cs2CdCl4:Sb3+ Ruddlesden–Popper Phase Nanoplatelets
Source: ACS Nano. 2021 Oct 20;15(11):17729–37. doi: 10.1021/acsnano.1c05684 (PMC8613908; doi:10.1021/acsnano.1c05684)
Supplement: Supplementary file 1 — nn1c05684_si_001.pdf [file nn1c05684_si_001.pdf]

Supporting Information

Cyan Emission in Two-Dimensional Colloidal $Cs_2CdCl_4:Sb^{3+}$ Ruddlesden–Popper Phase Nanoplatelets

Federico Locardi,*,†,‡ Margarita Samoli,‡ Alberto Martinelli,‖ Onur Erdem,‡ Debora Vale Magalhaes,⊥ Sara Bals,⊥ Zeger Hens,*,‡

†Department of Chemistry and Industrial Chemistry, Università degli Studi di Genova, Via Dodecaneso 31, 16146 Genova (Italy)

‡Physics and Chemistry of Nanostructures group (PCN), Ghent University, Gent 9000, Belgium

‖CNR-SPIN Corso Perrone 24, I-16152 Genova, Italy

⊥Electron Microscopy for Materials Science (EMAT), Department of Physics, University of Antwerp, Groenenborgerlaan 171, 2020, Antwerp, Belgium

- **Synthesis optimization** **S2**
- **Structural data** **S4**
- **XRD and TEM of the undoped sample** **S5**
- **Crystallite determination and X-ray total scattering** **S5**
- **Additional TEM images** **S6**
- **Additional ABS and PLE data** **S7**
- **TEM, ABS, PL and PLE after 6 months** **S8**

Synthesis optimization

The optimum experimental conditions used for the synthesis were achieved through several experiments. Following the influence of the different parameters are listed.

Benzoyl chloride: we noticed that a large amount of Cl^- (about 7 times the stoichiometric amount) was needed for having an emissive pure phase sample. When the amount of Bz-Cl was lower than 200 μL no precipitated was formed. The yield and the formation of an emissive product occurred by increasing the Bz - Cl (250 – 300 – 350 μL) even if the formation of CsCl as by products was detected. Only using 400 μL led to pure phase sample whose emission decreasing further increasing the Cl^- amount.

Ligands: the ligands amount and ratio are probably the two most important factors affecting the synthesis of our material (Figure S1). We noticed that even small variation (5%) of the used quantity strongly influenced the properties of the final product. Thus, the quantity of ligands employed were measured according to their weight and not volume (*i.e.* using a balance and not a micropipette); however, for a simpler comparison between the experiment, we also report the corresponding volumes.
The explored interval ranged between 1.3300 g (~ 1.5 ml) and 0.88 g (~ 1 ml) for OLAc and 1.220 g (~ 1.5 ml) and 1.60 g (~ 2 ml) for OLAm; hence, the OLAm was always in excess respect to OLAc, necessary conditions for having an emission in the synthesized materials. We noticed a systematic increased of sample emission increasing the OLAm/OLAc ratio ascribing to both an improvement in the phase purity and morphology. The maximum emission was reached using 1.4575 g (~ 1.8 ml) of OLAm and 1.0403 g of OLAc (~ 1.2 ml). Interestingly, further increasing the OLAm/OLAc ratio quenched completely the emission and CsCl was the only product detected. A possible explanation is that the different ratio employed influences the particles thickness, reducing it gradually till a minimum after which the Ruddlesden - Popper phase is no longer stable.
Finally, we noticed that the synthesis was completely reproducible only using OLAm stored under nitrogen.

Cs_2CO_3: increasing the Cs amount (+20%) induced the formation of CsCl as secondary phase; on the contrary, its reduction (-20%) preserved the purity of the phase but with worst emissive properties.

Temperature: it is well known that for metal halide nanocrystals the temperature mainly affects the size of the particles. Indeed, we noticed an increasing of the particle size plus a lowered homogeneity when the temperature of the injection increased above 100 °C. Under this value no precipitated was obtained (Figure S1).

$Sb(ac)_3$: tuning the amount (± 10% and ±20%) of Sb^{3+} employed during the synthesis lowered the general photoluminescence intensity (Figure S2).

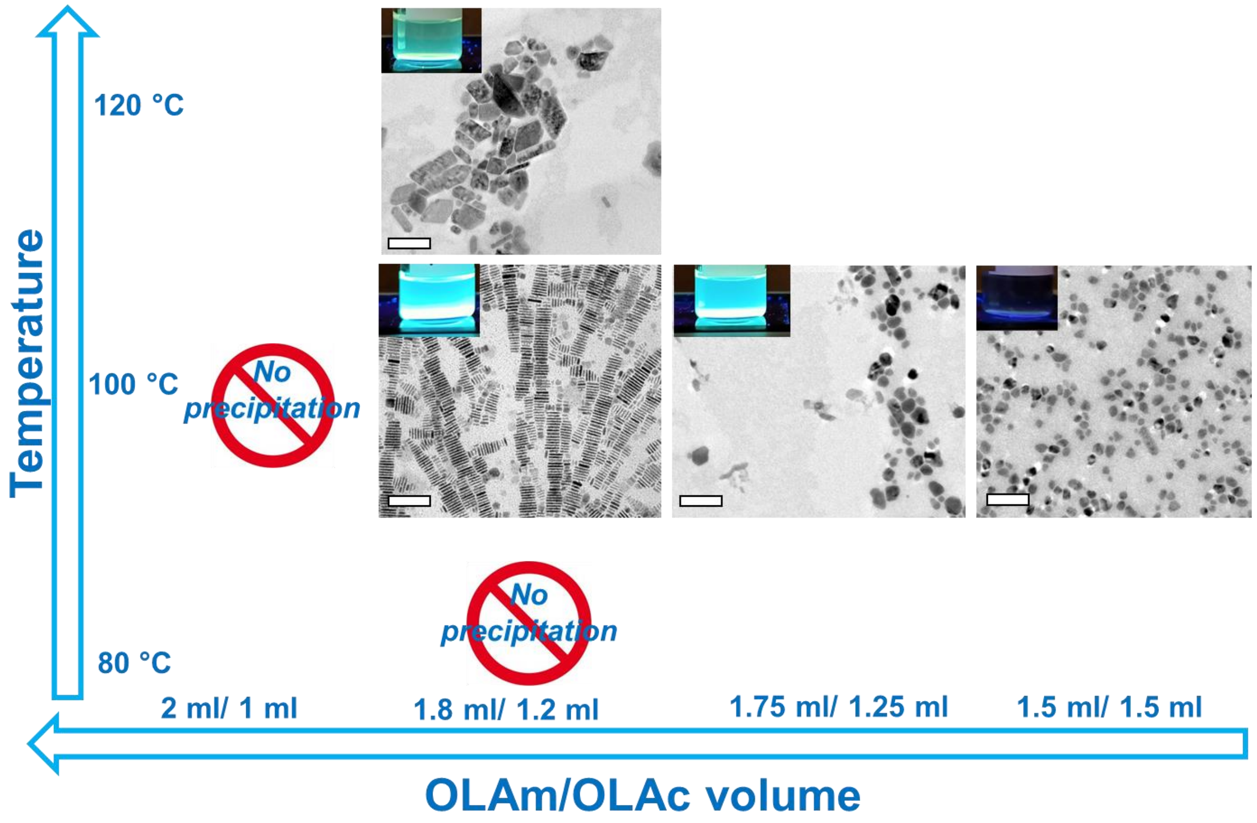

Figure S1. Effect of temperature and ligands on the $Cs_2CdCl_4:Sb^{3+}$ preparation. Homogenous nanoplatelets, with a considerable emission, were obtained using a precise OLAm/OLAc ratio and injecting the Bz-Cl at 100°C. The variation of these parameters has a detrimental effect on both morphology and emission.

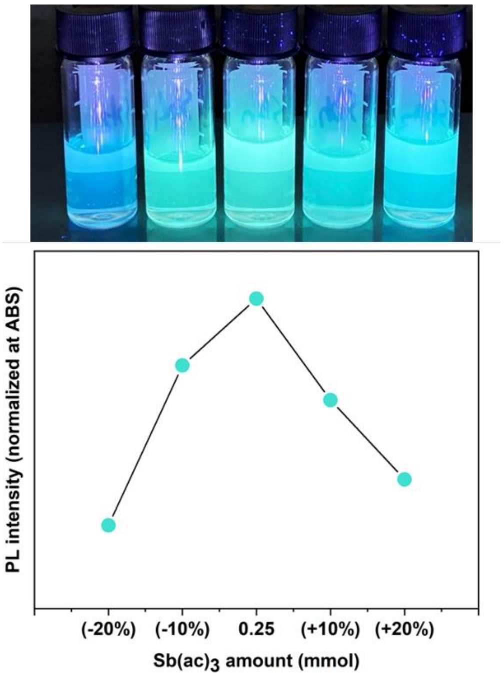

Figure S2. Effect of $Sb(ac)_3$ amount used during the synthesis on the photoluminescence intensity (normalized at the ABS value at $\lambda = 320$ nm).

Table S1. Structural data for the asymmetric unit of Cs_2CdCl_4; space group *I*4/*mmm* (n° 139).

**cell parameters**		*a* (Å)		5.2568(1)
		c (Å)		16.876(1)
**atomic positions**				
atom	Wyckoff site	*x*	*y*	*z*
Cs	4*e*	0	0	0.3676(1)
Cd	2*a*	0	0	0
Cl_1	4*c*	0	½	0
Cl_2	4*e*	0	0	0.1551(7)
**agreement factors**		R_{Bragg} (%)		5.40
		R_{factor} (%)		3.42

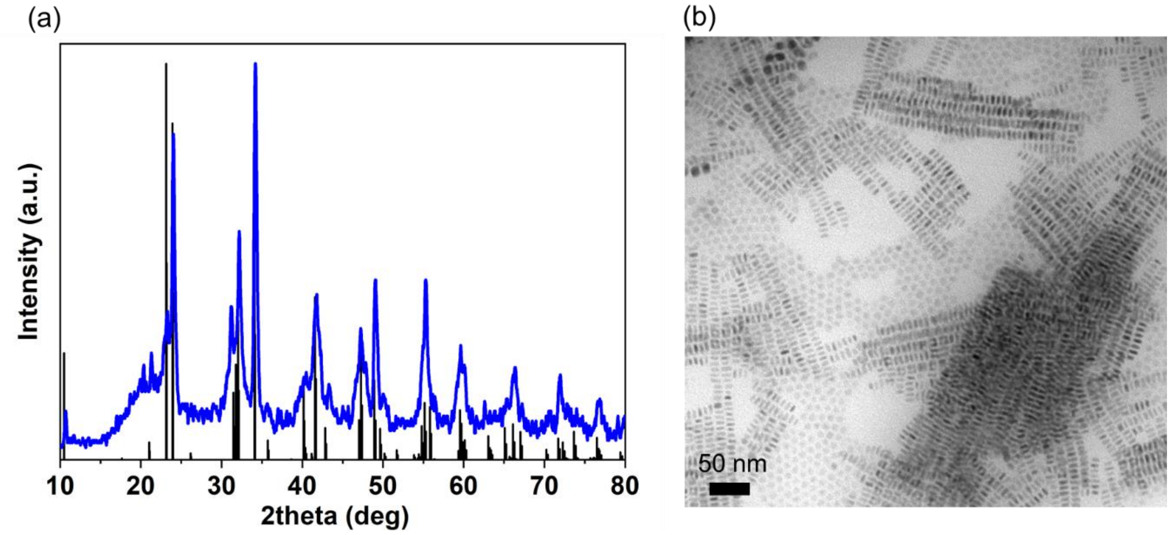

Figure S3. (a) XRD pattern and (b) TEM image of the undoped Cs_2CdCl_4 sample.

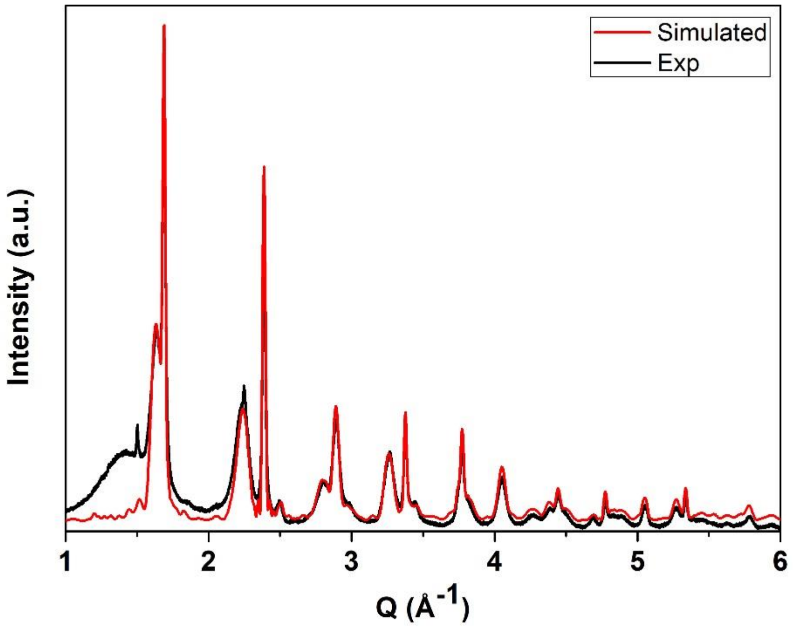

Figure S4. Experimental and simulated diffraction pattern calculated using the software DIANNA, considering the Cs_2CdCl_4 (space group *I*4/*mmm*; n° 139) structure and a crystallite dimension of 44 x 44 x 3 cells (equal to of 23 x 23 x 5 nm^3).

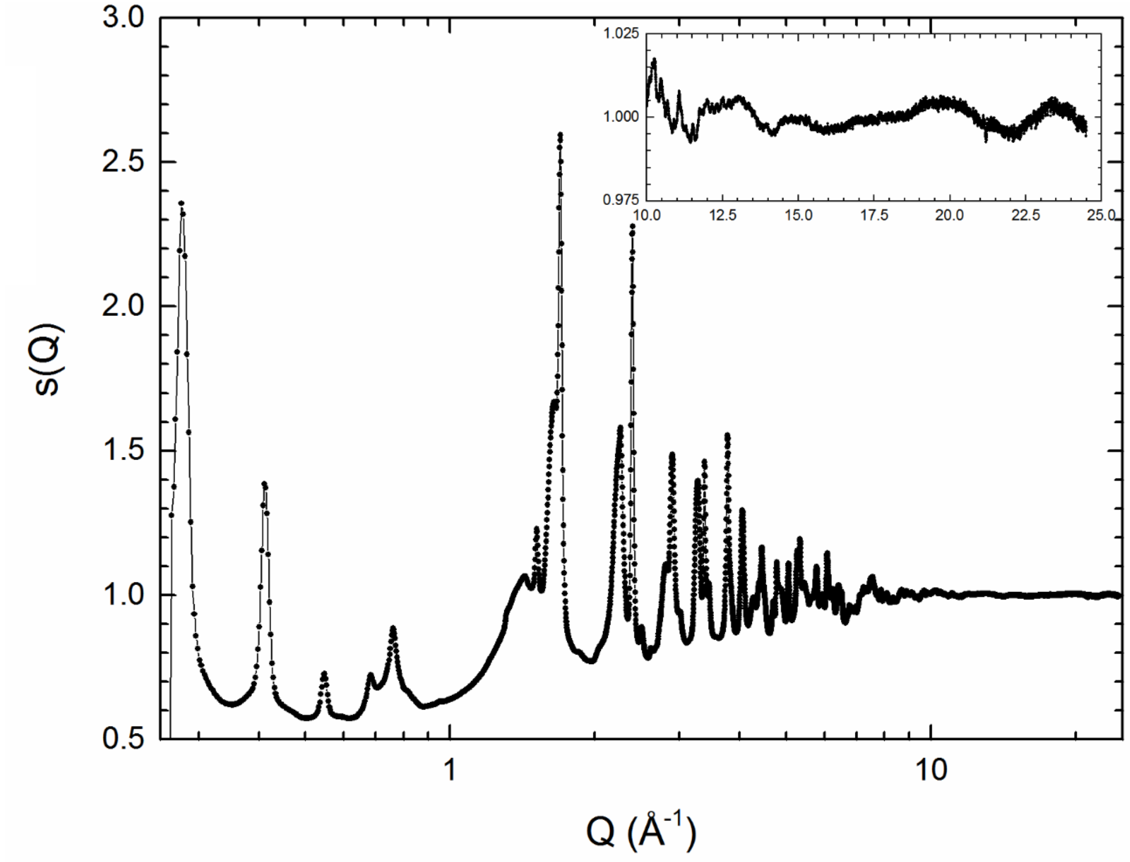

Figure S5. X-ray total scattering *S*(*Q*) function of Cs_2CdCl_4:Sb^{3+}, showing well-defined oscillations at high Q (inset), produced by the dynamic disorder affecting the frameworks of linked octahedra.

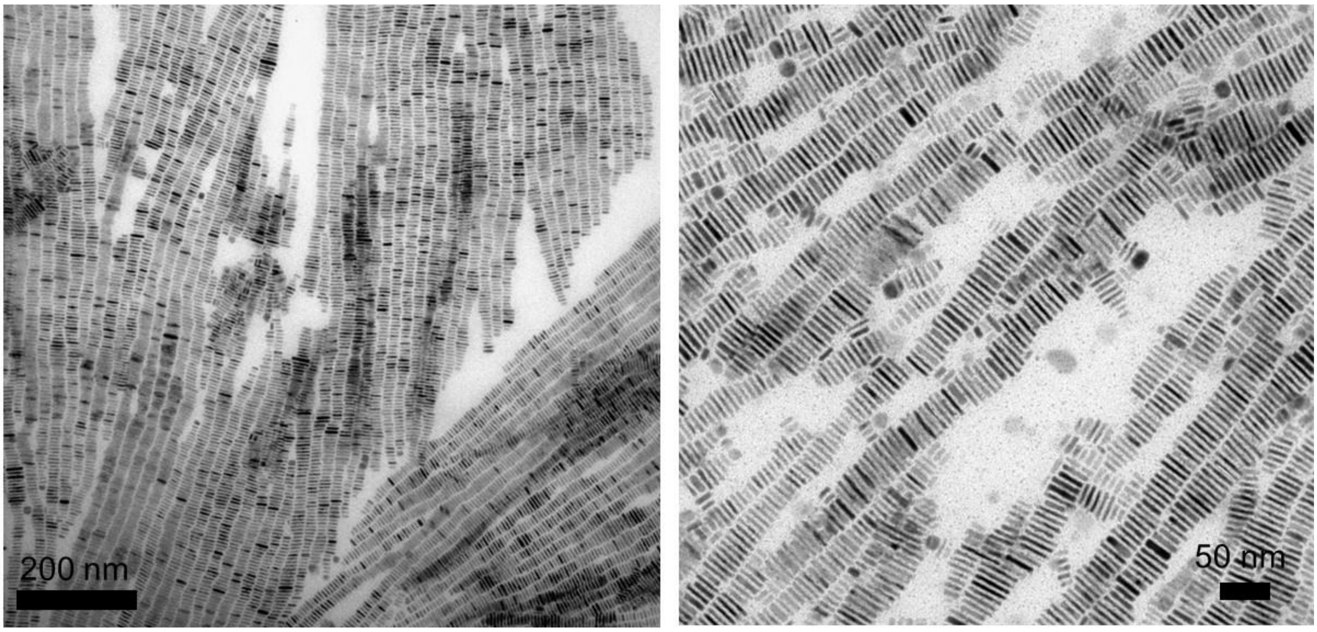

Figure S6. TEM images of Cs_2CdCl_4:Sb^{3+} that show the natural tendency of the nanoplatelets to assemble in long chains, stacking along the *ab* face.

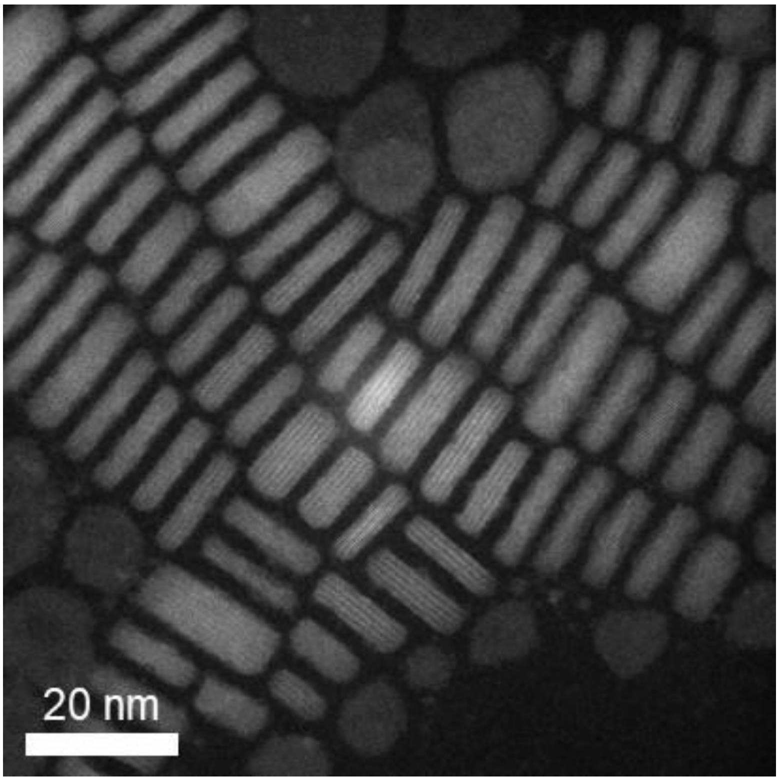

Figure S7. HAADF-STEM image overview of the Cs_2CdCl_4: Sb^{3+} nanoparticles.

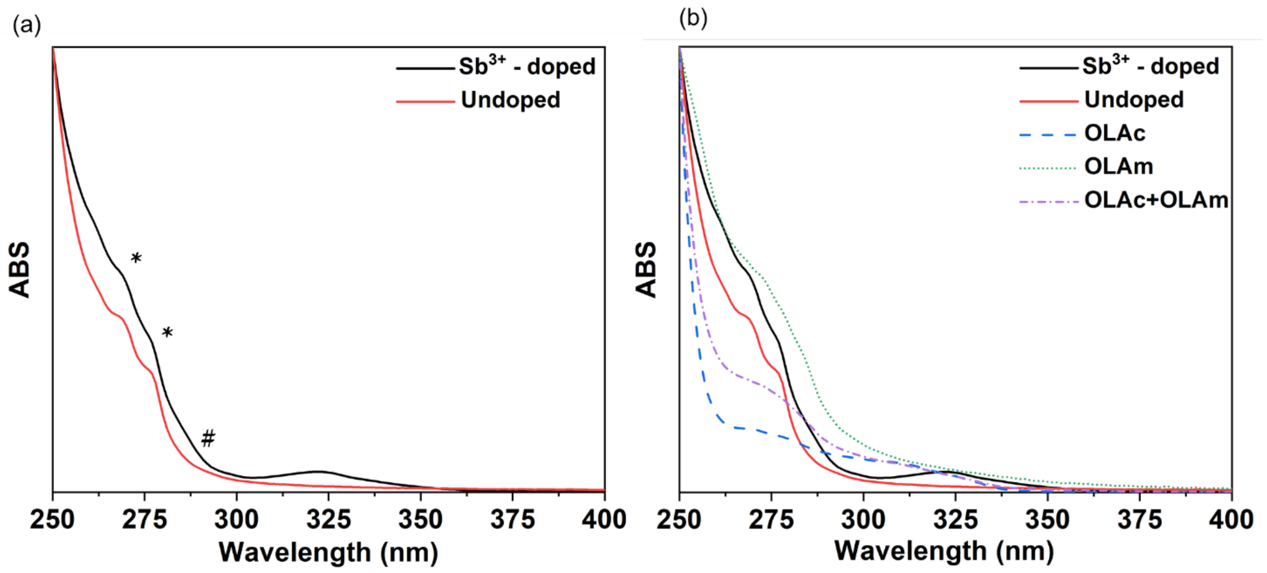

Figure S8. (a) ABS of the undoped (red) and Sb^{3+} - doped (black) Cs_2CdCl_4 samples and (b) comparison with the oleic acid and oleylamine absorption (5 μL in 3ml of hexane). The marked features were ascribed to the matrix and ligands (*) and to the C band of Sb^{3+} (#).

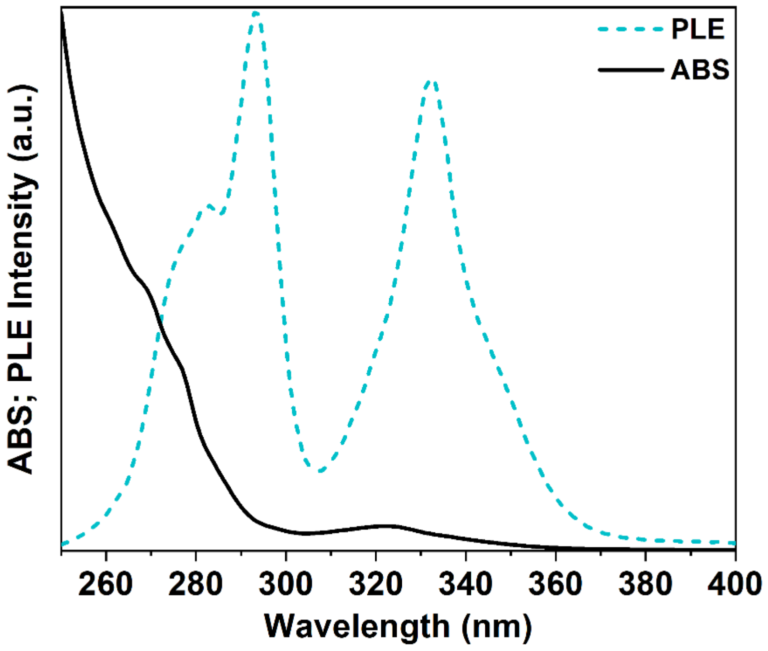

Figure S9. ABS and PLE of $Cs_2CdCl_4:Sb^{3+}$ NPs. The accordance between ABS and PLE is evident as the peaks split, induced by the distortion at local scale.

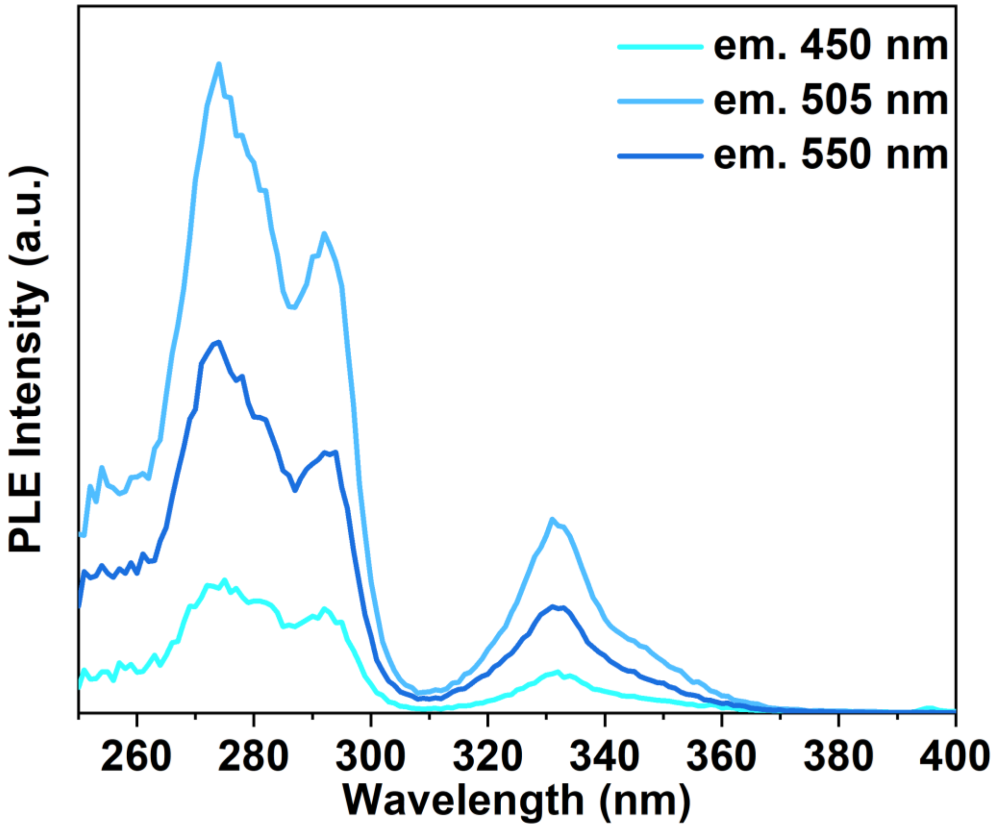

Figure S10. PLE excitation spectra of $Cs_2CdCl_4:Sb^{3+}$ collected at different emission wavelengths.

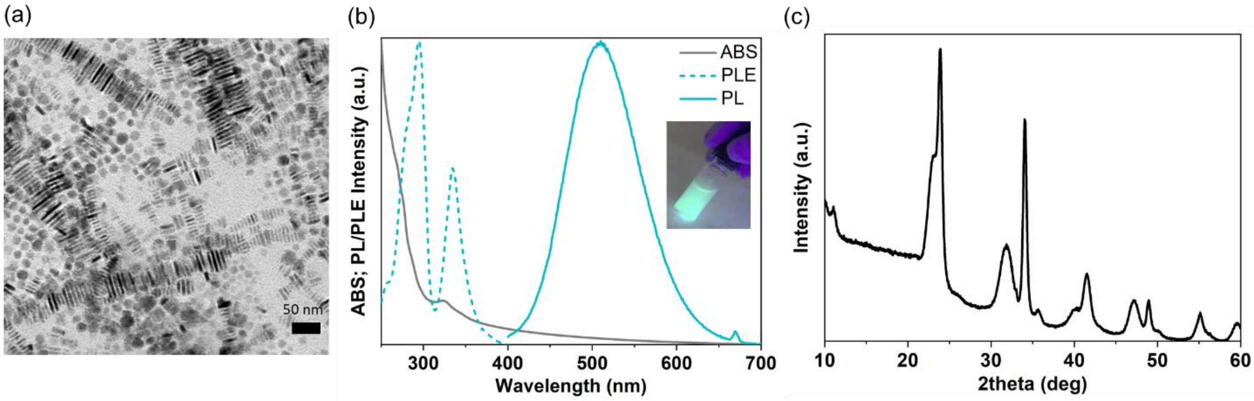

Figure S11. (a) TEM, (b) ABS, PL and PLE spectra and (c) XRD of $Cs_2CdCl_4:Sb^{3+}$ after 6 months of storage in a closed vial under air.
